# Unveiling the Potential of Three Endemic *Gypsophila* L. (Caryophyllaceae) Taxa as Promising Carbonic Anhydrase Inhibitors—Bio-Metabolic Profiles and In Vitro Evaluation of Enzyme Inhibition and Antioxidant Capacity

**DOI:** 10.3390/antiox14020219

**Published:** 2025-02-14

**Authors:** Eda Büker, Ayşenur Kayabaş Avşar, Ertan Yildirim, Dorina Casoni, Simona Codruța Aurora Cobzac, Claudia Cimpoiu

**Affiliations:** 1 Department of Basic Pharmaceutical Sciences, Faculty of Pharmacy, Gazi University, 06330 Ankara, Türkiye; edabiyik@gazi.edu.tr; 2 Department of Biology, Faculty of Science, Çankırı Karatekin University, 18100 Çankırı, Türkiye; aysenurkayabas@karatekin.edu.tr; 3 Department of Chemistry, Faculty of Science, Gazi University, 06500 Ankara, Türkiye; ertan.yildirim@gazi.edu.tr; 4 Department of Chemistry, Faculty of Chemistry and Chemical Engineering, Babeş-Bolyai University, 11 Arany János, 400028 Cluj-Napoca, Romania; dorina.casoni@ubbcluj.ro (D.C.); simona.cobzac@ubbcluj.ro (S.C.A.C.); 5 Research Centre for Advanced Chemical Analysis, Instrumentation and Chemometrics–ANALYTICA, Babeş-Bolyai University, 11 Arany Janos, 400028 Cluj-Napoca, Romania

**Keywords:** bio-metabolic profile, in vitro diuretic activity, endemic *Gypsophila* taxa, rosmarinic acid, antioxidant capacity, ATR-FTIR

## Abstract

The Caryophyllaceae family, commonly utilized in traditional medicine, exhibits various effects revealed by ethnopharmacological studies. Thus, the diuretic effect of the leaf and stem of three *Gypsophila* taxa endemic to Türkiye was evaluated for the first time by comparing their bio-metabolic profiles, antioxidant capacities, carbonic anhydrase inhibition, and infrared spectra. The leaf and stem of *Gypsophila* taxa were macerated in 50% ethanol and 50% water, bio-metabolic profiles were performed by a new validated ultra-performance liquid chromatographic (UPLC) method and spectrophotometric methods, the antioxidant capacity was determined by DPPH and ABTS assays, and the in vitro diuretic activity was evaluated by carbonic anhydrase inhibition. The results show that the *G. simonii* leaf exhibited the highest quantity of rutin and total polyphenols content (TPC). On the other hand, the *G. germanicopolitana* leaf showed the highest quantity of rosmarinic acid, and the *G. eriocalyx* leaf contained the maximum total flavonoids content (TFC). The antioxidant results indicated that *G. eriocalyx* has the highest capacity. The *G. germanicopolitana* leaf strongly inhibited the enzyme activity. The ATR-FTIR spectra showed that the general chemical composition in the leaf and stem parts was preserved after the extraction process. Band intensity changes may be due to the extraction process and the amount of substances. In conclusion, the species of *Gypsophila* taxa show considerable potential for utilization in the pharmaceutical area.

## 1. Introduction

Representatives of the Caryophyllaceae, one of the major families of Angiosperms, are cosmopolitan worldwide and are widely known as garden plants, but their medicinal importance is less well known [1]. *Gypsophila* L., the fourth largest genus of Caryophyllaceae with 37 genera in Türkiye, comes after *Silene* L., *Dianthus* L., and *Minuartia* L. in terms of the number of taxa [2]. Slightly more than 50% of *Gypsophila* species worldwide are found in Türkiye [3]. *Gypsophila* is represented by 67 taxa in Türkiye [2], 40 of which are endemic, giving an endemism rate of ~60%. *Gypsophila eriocalyx* Boiss., whose vernacular name is ‘bozkırçöveni’, is a perennial with a woody caudex, an Irano-Turanian floristic element. *Gypsophila germanicopolitana* Hub.-Mor., whose vernacular name is ‘Çankırıçöveni’, is a perennial with a woody rhizome, an Irano-Turanian floristic element. *Gypsophila simonii* Hub.-Mor., whose vernacular name is ‘Kalecikçöveni’, is a perennial with a thick rhizome, an Irano-Turanian floristic element [4,5].

The human body comprises approximately 60% water, and this vital substance plays a critical role in numerous physiological processes [6]. It is essential to maintain a balance of fluids and electrolytes within the body, as any disruption can have serious implications. Various mechanisms, such as the neural regulation of thirst, hormonal control [7] through vasopressin [8] and natriuretic peptides, management through the skin, hemodynamic adjustments, and the renal regulation [9] of salt and water excretion, work together to tightly regulate the body’s overall fluid balance [10].

The process of renal excretion involves the removal of excess water, metabolic waste products, and surplus electrolytes from the body. This process is crucial for maintaining the balance of fluids within the body [11], and the kidneys play a crucial role in this process. Frequently, diuretics are prescribed to manage conditions such as high blood pressure, heart failure, and edema [12].

Medications derived from plant origins represent a significant natural reservoir of compounds with potential for the development of innovative pharmaceuticals. These plant-based remedies have historically demonstrated efficacy in the treatment of both human and animal conditions, devoid of adverse effects [13]. Consequently, there is a concerted effort to explore and identify new medicinal plants as a means to engender more potent and cost-effective therapeutic agents in lieu of synthetic pharmaceuticals. Ayurveda, Traditional Chinese Medicine, and Traditional Ottoman Medicine are systems rooted in the use of medicinal plants. These traditional practices have extensively examined the therapeutic properties of herbs and plants, particularly in the context of various diseases such as hypertension and edema. *Gypsophila* taxa have been studied and used among the medicinal plants in Traditional Chinese Medicine and Iranian culture for a long time as anticancer, antibacterial, antidiuretic, antifungal, antiviral, antioxidant, and anti-inflammatory drugs [14]. These ethnopharmacological studies reveal that members of this family exhibit such properties. Biomedical investigations on *Gypsophila* taxa need to be conducted, with a focus on identifying the active principles associated with various activities. Additionally, *Gypsophila* has been a commonly studied genus in both ethnomedicinal and pharmaceutical research; however, the biomedical properties of other genera within the family warrant further exploration. Given the diverse and promising biomedical activities observed, it is essential to pursue further studies on drug development utilizing different plant extracts and their constituents. Both traditional medicine’s safety and accepted applications and endemic *Gypsophila* taxa have not been examined in terms of their diuretic effects, so this study was focused on unveiling the effect of endemic *Gypsophila* taxa on diuresis.

Polyphenols are secondary plant metabolites that can be classified according to their structure into four main groups, among which flavonoids and phenolic acids are the most known. The literature shows many medicinal effects of these compounds, including adiuretic effect [15]. Polyphenols can be identified and quantified individually by chromatographic methods such as high-pressure liquid chromatography (HPLC). Spectrophotometric methods can be also employed for determining the total polyphenols content (TPC), but they are not specific to all polyphenols being evaluated.

Flavonoids are a wide-ranging collection of natural compounds characterized by their phenolic structure. They are present in a variety of plant sources, such as many kinds of food. Their plant sources have been recognized for their health benefits since ancient times, and researchers have identified more than 4000 different types of flavonoids, which are responsible for the attractive colors of flowers, fruits, and leaves [16].

Chemical analyses are very important for monitoring biological processes in nature. In addition to chromatographic analyses, information about important chemical compositions can also be obtained from the chemical analysis of plant species in nature. Fourier-transform infrared (FT-IR) spectroscopy, which is a fast, reliable, and inexpensive method, is used to determine the functional chemical groups of compounds found in herbal extracts [17]. In addition to the molecular analysis of plant extracts, information about both qualitative and quantitative chemical analyses can be determined from the bands of functional groups in the ATR-FTIR spectra. Band types and intensities belonging to functional chemical groups also shed light on chromatographic studies.

The literature suggests that extracts obtained from plant materials contain a wealth of bioactive compounds with substantial antioxidant properties [18]. Research focused on assessing the antioxidant capacity of diverse plant species holds the potential to underscore the significance of these species as a rich source of antioxidants, thereby enhancing their potential therapeutic utility. The methods of measuring the antioxidant capacity of natural samples show extreme diversity. Most of the methods are based on measurements involving various free radicals, while other measurements include reactions with different metal ions (for example, FRAP assay or CUPRAC assay) [19]. Methods based on the scavenging of the free-radicals 2,2-diphenyl-1-picrylhydrazyl (DPPH•) and 2,2′-azinobis-(3-ethylbenzothiazoline-6-sulfonic acid) (ABTS•+) were found to be used mostly for the in vitro antioxidant capacity assessment goal [20].

Considering all the presented aspects, this study focused on examining the hydroalcoholic extracts from the leaf and stem parts of three *Gypsophila* taxa native to Türkiye (*G. eriocalyx*, *G. germanicopolitana*, and *G. simonii*)in terms of in vitro diuretic activity. This study also aimed to assess the correlation between the in vitro diuretic activity, antioxidant capacity, and quantities of polyphenols compounds. Additionally, this research also explored the IR spectra of the hydroalcoholic extraction samples to reveal the effectiveness of the extraction procedure on the active compounds.

## 2. Materials and Methods

### 2.1. Reagents and Equipment

The solvents were of UPLC purity and were purchased from Merck (Darmstadt, Germany). The standards were acetazolamide, carbonic anhydrase enzyme, *p*-nitrophenyl acetate, rutin, rosmarinic acid, and quercetin and were purchased from Thermo Scientific Chemicals (Darmstadt, Germany).

The target compounds were chromatographically quantified using the state-of-the-art ACQUITY UPLC H-Class instrument (Waters, MA, USA). This advanced instrument is specifically equipped with a quaternary solvent manager to precisely regulate the solvents used in the chromatographic process. It also includes a highly sensitive UV detector to detect and analyze the separated compounds accurately. In addition, the instrument features a cooling autosampler to maintain sample integrity and an integrated oven for precise control of the column temperature, ensuring optimal chromatographic performance. The Waters × Bridge 4.6 × 50 mm column (50 mm × 2.1 mm i.d., 1.7 mm) was employed for the elution of the related active compounds. The quantification of rosmarinic acid, rutin, and quercetin was carried out using the Waters^®^ Empower 3.8.0 software. UV spectrum measurements were conducted in the range of 200–500 nm wavelength using a Jasco V-550 UV spectrophotometer (Jasco Corporation, Tokyo, Japan). Spectra Manager for Windows 95/NT version 1.53.04 (1995–2002, Jasco Corporation) software package was used for the spectra acquisition control, smoothing process, storage and spectral data digitization. The milling procedure to achieve extractions was executed with Retsch MM400 ball mill (Retsch, Haan, Germany), and the compounds were weighed with a Sartorius CPA225D-OCE balance (Sartorius group, Göttingen, Germany).

The ATR-FTIR spectroscopy system used in the chemical analysis of plant extracts is the Thermo Nicolet 6700 model and is supported by OMNIC software 6.2 (Thermo Fisher Scientific Inc., Waltham, MA, USA). The spectra of plant extracts were obtained at room temperature with a resolution of 4 cm^−1^, 64 spectrum scans, and wave numbers of between 400 and 4000 cm^−1^.

### 2.2. Plant Material

The leaf and stem parts of three endemic *Gypsophila* taxa (*G. eriocalyx*, *G. germanicopolitana*, and *G. simonii*) were examined. The study materials were collected from five adult individuals from the natural population of *Gypsophila* taxa growing in the steppe vegetation of the Çankırı-Ankara road Ballıca Campus locality (630–670 m a.s.l., 40°31′ N, 33°36′ E, Çankırı province, Türkiye). The selection of plants was based on criteria such as living in the same climatic and edaphic conditions, showing morphologically similar architectural features, and perennial life strategies. According to the Turkish *Red Data Book*, root samples were not taken from *G. eriocalyx*, *G. germanicopolitana*, and *G. simonii*, which show LR (lc), CR, and VU features according tothe IUCN threat categories [21], respectively; only stem and leaf samples were collected from plants growing in their natural habitats. In order to minimize chemical variability originating from differences in the phenological stages of plants, the stem and leaf samples were collected in late June, which coincides with the end of the vegetation period. Taxonomical identifications of the *Gypsophila* taxa were made according to the *Flora of Turkey and the East Aegean Islands* [4]. The identification of plant species was conducted by the author (A. Kayabaş Avşar). Voucher materials were deposited as a personal collection at the Çankırı Karatekin University.

### 2.3. Maceration of Plant Samples

The plant parts were ground into powder with an herb grinder, and then the powdered samples were milled. In the cold maceration step, 4 g milled leaf and stem samples of each *Gypsophila* taxa were weighed and transferred into a glass tube filled with a 40 mL solvent mixture, which consisted of ethanol and water (50:50, *v*/*v*). All glass tubes were kept in the dark at room temperature for ten days, and the mixtures were mixed daily. After ten days, all six extracted samples were filtered with a 0.45 µm syringe filter. The filtered extracts were set aside at 4 °C for further analysis, namely, UPLC analysis, antioxidant capacity assays, and in vitro diuretic analysis.

### 2.4. Chromatographic Analysis

All extracts were analyzed with the following UPLC conditions: Waters × Bridge 4.6 × 50 mm column (50 mm × 2.1 mm i.d., 1.7 mm), mobile phase A was 0.1% formic acid in water, mobile phase B was 0.1% formic acid in acetonitrile (can), and the ratio between these two mobile phases was 80% of A and 20% of B. The flow rate was 0.45 mL/min, and the temperature was 45 °C. The injection volume was 10 µL, and the detection wavelength was 260 nm.

The stock solutions for the analyzed compounds, including rosmarinic acid, rutin, and quercetin, were prepared by dissolving 10 mg of each compound in 100 mL of absolute methanol. Additionally, a training set consisting of 5 different mixture samples containing varying concentrations of rosmarinic acid, rutin, and quercetin was made by diluting each standard solution in methanol. These mixtures were prepared to obtain concentrations ranging from 8.0 to 100.0 µg/mL for rosmarinic acid, 0.5 to 10 µg/mL for rutin, and 5.0 to 40.0 µg/mL for quercetin. A set of test samples containing synthetic binary mixtures was prepared using stock solutions of compounds. These mixtures were set up using a range of concentrations within the linear working range of the relevant drugs.

To thoroughly verify the accuracy and precision of the methods, samples for both intra-day and inter-day experiments were methodically taken. These samples were carefully prepared at three distinct concentrations for rosmarinic acid and quercetin: 10 µg/mL (first grade), 20 µg/mL (second grade), and 30 µg/mL (third grade). Similarly, for rutin, the concentrations used were 2 µg/mL (first grade), 6 µg/mL (second grade), and 10 µg/mL (third grade). Each concentration was accurately prepared using the corresponding stock solutions to ensure accuracy and consistency. These specific concentrations were chosen to thoroughly investigate the performance of the methods across a range of levels.

### 2.5. In Vitro Evaluation of Carbonic Anhydrase Inhibition

The methodology used for in vitro diuretic activity determination was based on the research conducted by Armstrong et al. [22], with certain adjustments. In total, 3 mM of p-nitrophenyl acetate (substrate) was dissolved in a mixture of 0.3 mL acetone and 9.7 mL deionized water. The carbonic anhydrase enzyme solution was prepared by dissolving 2.71 mg of the enzyme in 2 mL of phosphate buffer (pH 7.0). The solution of the standard inhibitor (acetazolamide) was prepared with a concentration range of between 1 and 76 ppm in methanol. The substrate sample was prepared as a mixture of 0.5 mL of substrate, 0.65 mL of phosphate buffer (pH 7.0), and 1.02 mL of deionized water. A mixture of the substrate and enzyme sample was prepared by mixing 0.5 mL of substrate solution, 0.65 mL of phosphate buffer, 20 µL of enzyme solution, and 1.0 mL of deionized water. An inhibitor sample, which was obtained from extraction, was prepared as a mixture of 0.5 mL of substrate solution, 0.65 mL of phosphate buffer, and 20 µL of enzyme solution filled with 2.17 mL of inhibitor solution. Amixture of 0.33 mL of acetone and 9.7 mL of deionized water was used as a blank sample. All measurements were conducted at a wavelength of 400 nm and a temperature of 25 °C, utilizing a Jasco V-550 spectrophotometer (Jasco Corporation, Tokyo, Japan). Acetazolamide was used as standard, and % of inhibition was calculated by the following formula:

% Inhibition = [(A_Blank_−A_Sample_)/A_Blank_] ×100, (1)

where A_Blank_ is the absorbance value for the blank solution, and A_Sample_ is the absorbance value for the sample.

As reported in [23], the diuretic activity was quantified through efficient concentration parameters (IC_50_), which were determined using a linear regression equation derived from the relationship between the percentage of inhibition and the volume of the added extract.

### 2.6. Total Flavonoid Content (TFC)

Total flavonoid content (TFC) was determined by using the spectrophotometric AlCl_3_ method [24]. Volumes of 0.5 mL of hydroalcoholic extract diluted (1:10 *v*/*v*) with ethanol–water (50:50, *v*/*v*) were mixed with 2.5 mL of AlCl_3_ solution (100 g/L) and 1.5 mL of CH_3_COONa solution (25 g/L) and brought up with methanol to 10 mL (volumetric flask). After 15 min, the absorbance was measured at 430 nm. The reference solution was prepared in the same manner but using water (4 mL) instead of aluminum chloride and sodium acetate solutions. The TFC was reported as mg of rutin equivalents (REs)/mL. The rutin calibration curve was plotted in the range of 1–50μg/mL by following the protocol described above.

### 2.7. Total Polyphenols Content (TPC)

Total polyphenols content (TPC) was determined spectrophotometrically by the Folin–Ciocalteau method [25]. Volumes of between 100 and 1000μL of hydroalcoholic extract diluted (1:10) with ethanol–water (50:50, *v*/*v*) were mixed with 0.5 mL of Folin–Ciocalteau reagent and brought to 25 mL (volumetric flask) with Na_2_CO_3_ solution (16%). Absorbance was measured at 715 nm after 30 min. The reference solution was prepared in the same way as the sample but without adding the reagent. TPC was reported as mg of gallic acid equivalents (GAEs)/mL. The calibration curve was plotted in the range of 0.234–4.672 μg/mL.

### 2.8. Antioxidant Capacity

The antioxidant capacity of *G. eriocalyx*, *G. germanicopolitana*, and *G. simonii* extracts was determined by spectrophotometric assay using 1,1 diphenyl-2-picrylhydrazyl (DPPH•) and 2,2′-azino-bis(3-ethylbenzothiazoline-6-sulfonic acid) (ABTS^+^•) radicals.

#### 2.8.1. DPPH Radical Scavenging Capacity Assay

The DPPH• assay [26] with some minor modifications was used for the evaluation of antioxidant capacity. To maintain the free radical activity, the solution was freshly prepared each day and protected from light for the entire duration of the analysis. In all cases, 4 mL of DPPH•ethanolic solution of 0.15 mmol/L was mixed with different volumes of extract (volume range of the added extract was 50 μL–500μL) and then brought to a final volume of 5 mL with ethanol. The absorbance values were measured at a λ = 518 nm wavelength after a reaction time of 60 min in dark conditions. In all cases, the percentage of consumed radicals was determined using the following equation:

Neutralized radical (%) = [(A_0_−A_f_)/A_0_] × 100, (2)

where A_0_ is the absorbance of the DPPH• radical without the addition of the plant extract; A_f_—final absorbance of the radical solution after 60 min of reaction with the added extract.

The assessment of antioxidant capacity utilized the efficient concentration (EC_50_) parameter, denoting the volume of extract (in µL) required to diminish the initial concentration of the DPPH• solution by 50%. In all cases, the EC_50_ parameter was determined from the linear regression equation obtained from the representation of the percentage of consumed DPPH• vs. the added volume of the extract. The EC_50_ values demonstrate an inverse correlation with the antioxidant capacity of the sample, with lower EC_50_ values unequivocally indicating heightened antioxidant capacity within the extract.

#### 2.8.2. ABTS Radical Scavenging Capacity Assay

In this assay, an ABTS^+^• radical stock solution was prepared. In line with the Ozcan protocol [27], the ABTS stock solution was prepared by combining equal volumes of a 7 mmol/L concentration of ABTS with a solution of 2.45 mmol/L potassium persulfate. After mixing, the solution was kept in the dark at room temperature for 12–16h before use. The working solution was obtained by diluting it with water until an absorbance of 0.7 ± 0.05 was reached. Aliquots of 4 mL of this solution were mixed with different volumes of diluted 1:10 (*v*/*v*) hydroalcoholic extract and then brought to a final volume of 5 mL. After a reaction time of 60 min in dark conditions, the absorbance was measured at λ = 752 nm. The percentage of consumed radicals was determined using Equation (3):

Consumed ABTS^+^• (%) = [(A_0_ − A_f_)/A_0_] × 100, (3)

where A_0_ is the absorbance of the ABTS^+^• solution (without the addition of plant extract); A_f_—final absorbance of the ABTS^+^• solution after 60 min of reaction with the added extract.

The antioxidant capacity was expressed as efficient concentration parameters (EC_50_), determined based on the linear regression equation obtained from the representation of the percentage of consumed ABTS**^+^•** vs. added volume of the extract.

#### 2.8.3. Ferric Reducing Antioxidant Power (FRAP) Assay

The FRAP assay was performed according to the procedure described by Scrob et al. [28]. Succinctly, the fresh working FRAP solution was prepared by mixing 2.5 mL of 0.01 M 2,4,6-tripyridyl-s-triazine (TPTZ) solution in 40 mM HCl, 2.5 mL of 0.02 M FeCl_3_·6H_2_O solution, and 25 mL of 0.3 M acetate buffer (pH = 3.6). The mixture was warmed at 37 °C. Aliquots of 150 µL of the extracts were mixed with 2850 µL of FRAP solution and incubated in the dark for 30 min. The absorbance of the resulting complex (Fe^3+^-TPTZ) was measured at 593 nm against a blank sample. Results were expressed as µg of ascorbic acid equivalents (AAEs)/mL.

### 2.9. Statistical Analysis

Principal component analysis (PCA) was applied for sample classification and to reveal the relationships between the determined parameters and the antioxidant and diuretic activity of the analyzed extracts. In this approach, the representation of the score plot of the first two principal components (PC1 vs. PC2) is a map of the analyzed samples. Samples close to each other have similar profiles, whereas those far from each other are dissimilar. Projection of the variables on the factor plane (loading value representation) was used for score interpretation and to reveal the correlations among the analyzed parameters and determined activities. The Statistica 12.0 software package (Stat Soft. Inc. 1984-2014, Tulsa, OK, USA) was used for the PCA analysis.

## 3. Results and Discussion

Although it is known in the literature that different plants exhibit different behaviors in terms of composition and biological activities, in different phenological stages, in the case of our studied plants, the flowering period coincides with a dry period due to the arid and semiarid climate characteristics of Çankırı province where some *Gypsophila* taxa grow. Since most gypsophile plants (gypsum-loving), such as some *Gypsophila* taxa, exhibit late flowering phenology [29], the coincidence of flowering stages with a dry period may also lead to the possibility of the plant being exposed to drought stress. Thus, the stem and leaf sampling was carried out in the late vegetation period, not in the flowering period when the plants start to dry rapidly, so that the obtained results are reproducible.

### 3.1. Chemical Analysis of Plant Extracts by ATR-FTIR Spectroscopy

**Figure 1:**
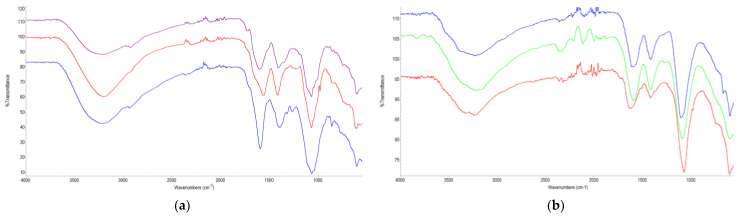
ATR-FTIR spectra of *Gypsophila* taxa: (**a**)—stem of *G. germanicopolitana* (purple), *G. simonii* (red), and *G. eriocalyx* (blue); (**b**)—leaf of *G. eriocalyx* (green), *G. germanicopolitana* (red), and *G. simonii* (blue).

Spectra were obtained using ATR-FTIR spectroscopy for the chemical analysis of the stem and leaf parts of the *Gypsophila* extracts (Figure 1).

In the spectra obtained from the stem (Figure 1a), O–H stretch bands in the stem of the *Gypsophila* taxa were detected at ~3650–3000 cm^−1^ and were referred to as gypsum. A high intensity of O–H stretching bands was detected in *G. simonii*. The highest was calculated in leaves, and the lowest was calculated in stems. At wave numbers ~2935 and ~2853 cm^−1^, where the aliphatic bands are intense, it was determined that the symmetric and antisymmetric CH_2_ stretching bands belong to the functional bands of oils, waxes, and lipids. After the extraction process, the lowest aliphatic band density was found in *G. simonii*. A carbonyl band belonging to esters was detected only in *G. germanicopolitana* at 1735 cm^−1^, but this band was not seen in the other stems. As a result of the extraction, the broad 1680–1580 C=C band, which is thought to originate from proteinic structures, was detected in *Gypsophila* taxa. In particular, stretch bands belonging to calcium oxalate were not seen in the stem parts of the *Gypsophila* taxa. Weak bands belonging to C=C double bands in lignin and phenolic structures were observed at 1515–1505 cm^−1^. The functional C–O stretching bands found in calcium carbonate were detected broadly at 1444–1370 cm^−1^. The intensities of these bands differed for the *Gypsophila* taxa after extraction. C–O stretching bands in the structure of calcium oxalate were found in the *G. eriocalyx* stem and *G. simonii* stem at around 1318–1820 cm^−1^. The C–O stretch bands belonging to phenolic and arylmethylethers in the lignin structure were detected at 1250–1235 cm^−1^. The highest stress intensity of this band was found in the *G. eriocalyx* stem. Broad bands originating from sulfate (S–O), silicate (Si–O), and phosphate (P–O) structures in the stem section were detected in the *Gypsophila* taxa at 1180–995 cm^−1^. C–O stretching and O–H deformation bands in polysaccharides were observed at approximately 1045 cm^−1^. The C–O band belonging to calcium carbonate was detected at 871 cm^−1^ in the stem parts of the *Gypsophila* taxa. COO bending bands of calcium oxalate were detected in the *G. eriocalyx* stem trunk at 780 cm^−1^. The *G. eriocalyx* and *G. simonii* stem S–O bending bands of *G. germanicopolitana* were determined at ~597 and ~669 cm^−1^.

All bands observed for functional chemical groups in the stem were also observed in the leaf (Figure 1b). The presence of specific bands also occurred in the leaf, and after the extraction process, the *Gypsophila* taxa preserved their chemical properties for the leaf and stem. The compatibility of the functional bands of these plant species with the ATR-FTIR spectra taken before extraction is an indication of the presence of basic compounds in the plants after the extraction process. The change in the intensities of the functional bands is due to the processes originating from the extraction process. At the same time, the changes in the intensities of the functional bands found in the plant species showed that the extraction process and the chemical composition of the stem and leaf were different in terms of quantity [17].

### 3.2. Total Flavonoid Content (TFC)

Flavonoids were quantified by using the AlCl_3_ spectrophotometric method, in which the reaction between AlCl_3_ and the phenyl groups of flavonoids occurs, leading to a yellow soluble complex, whose absorbance was measured at 430 nm.

To determine the content of flavonoids, a rutin calibration curve was plotted in the range of 1–50 μg/mL (Figure S3) on the basis of which the TFC was calculated (Table 1). The highest TFC was detected in the *G. eriocalyx* leaf extract. It was also observed that the leaf extracts contain much more flavonoids than the extracts of stems.

### 3.3. Total Polyphenol Content (TPC)

The TPC determined by the Folin–Ciocalteau method is presented in Table 1, the results being obtained based on the calibration curve of gallic acid in the range of 0.11–3.44 µg/mL (Figure S4). The results indicate that *G. simonii* leaf extract has the highest amount of polyphenols. Moreover, the same pattern is observed in the case of flavonoids—the leaf extracts contain more polyphenols than those of stems. Both the TFC and TPC results are in agreement with the data obtained on other plants with potential medical applications [30,31].

### 3.4. Chromatographic Analysis

Rosmarinic acid is a naturally occurring compound resulting from the esterification of caffeic acid and 3,4-dihydroxyphenyllactic acid and belongs to the family of hydroxycinnamic acids. Recent research on plants used in popular medicine revealed that rosmarinic acid has diuretic activity [32], leading to a K^+^ sparing effect. Rutin, also referred to as quercetin-3-O-rutinoside, is a type of flavonol glycoside that is widely recognized for its numerous health benefits. The molecule is composed of two main parts, namely, a phenolic part that is responsible for the antioxidant properties and a sugar part that makes the molecule more hydrophilic and soluble in water. When the molecule is broken down by the enzyme glucosidase, it produces two compounds: quercetin and rutinose. The literature shows that both rutin and quercetin have diuretic activity [33,34]. For these reasons, the determination of rosmarinic acid and rutin in *Gypsophila* taxa is an important step in revealing the diuretic effect of these species.

**Figure 2:**
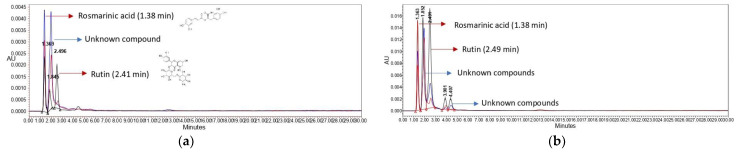
Chromatographic profile of the *Gypsophila* taxa: (**a**) stem extracts and (**b**) leaf extracts of *G. eriocalyx* (red), *G. germanicopolitana* (blue), and *G. simonii* (black).

Preliminary studies were performed with different column temperatures, a mixture of mobile phases, and different flow rates to evolve a new chromatographic method for the simultaneous analysis of rutin and rosmarinic acid in the extracts of *Gypsophila* species. The chromatographic profile at 260 nm of extracts obtained using the optimized UPLC-UV method is presented in Figure 2.

As can be seen from this figure, rosmarinic acid (1.38 min.) and rutin (2.41 min.) were detected in all *Gypsophila* extracts. Therefore, quantitation was performed only for these two compounds in the *Gypsophila* extracts. Before the analysis of rosmarinic acid, rutin, and quercetin, a mixture of rosmarinic acid, rutin, coumaric acid, quercetin, apigenin, and kaempferol (concentration of each compound was 20 ppm in methanol) was analyzed with the same chromatographic conditions to verify that the compounds did not overlap. The retention time of rosmarinic acid, rutin, coumaric acid, quercetin, apigenin, and kaempferol was 1.38 min, 2.41 min, 3.32 min, 12.42 min, 22.91 min, and 26.24 min, respectively (see Figure S1, Supplementary Materials). The newly developed chromatographic method was subjected to validation using test samples. The calculation was only conducted for rosmarinic acid and rutin in the *Gypsophila* extracts, but three compounds, rosmarinic acid, rutin, and quercetin, are quantified and detailed in Table 2 for the validation of the newly developed UPLC method. The UPLC conditions included mobile phases of A, 0.1% formic acid in water, and B, 0.1% formic acid in acetonitrile (80:20, *v*/*v*); a flow rate of 0.45 µL/min; a column temperature of 45°C; and an injection volume of 10 µL.

#### 3.4.1. Validity of the UPLC/UV Method

The validation of the newly developed UPLC method was performed using the quantification of three compounds, namely, rosmarinic acid, rutin, and quercetin (Table 2). In the current research, the accuracy and reliability of the UPLC/UV method were taken into consideration for validation as part of our analysis. This involved assessing the method’s ability to predict the quantities of rosmarinic acid, rutin, and quercetin in a variety of test samples, including binary mixtures, inter-day and intra-day samples, and standard addition solutions. We conducted comprehensive analyses utilizing the UPLC/UV method to measure the quantities of rosmarinic acid, rutin, and quercetin in different synthetic mixtures. The results of these analyses, along with the corresponding recovery values for both compounds, are documented in Table 2. Furthermore, to enhance the robustness of our findings, analyses of intra-day and inter-day samples containing rosmarinic acid, rutin, and quercetin were performed using the UPLC/UV method. These analyses yielded quantification results and recovery values, which are succinctly summarized in Table 3.

The calibration models demonstrated the ability to produce highly dependable, precise, and reliable analysis results for quickly evaluating the active compounds present in the test sample. The standard addition method is used to confirm the effectiveness of the UPLC/UV method and to assess how the presence of inactive components affects the analysis of extracts from three *Gypsophila* taxa. The experimental results, along with corresponding recovery values and relative standard deviations, are presented in Table 4.

The results effectively demonstrate the specificity and selectivity of the developed UPLC/UV method in quantifying the amount of rosmarinic acid and rutin in the analyzed extracts. In the examination of standard spiking solutions, it is found that the presence of other compounds does not have any effect on the accurate measurement of the target compounds.

#### 3.4.2. Quantification of Rosmarinic Acid and Rutin in Extraction Samples of *Gypsophila* Taxa

**Figure 3:**
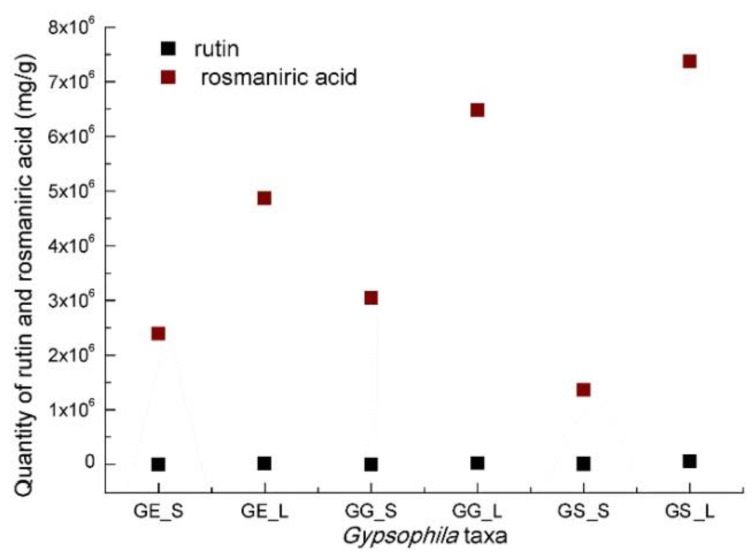
Quantification levels of rosmarinic acid and rutin in three different extracts of *Gypsophila* taxa: *G. eriocalyx* stem (GE_S), *G. eriocalyx* leaf (GE_L), *G. germanicopolitana* stem (GG_S), *G. germanicopolitana* leaf (GG_L), *G.simonii* stem (GS_S), and *G. simonii* leaf (GS_L).

The chromatograms of *Gypsophila* extracts were recorded to illustrate the relationship between the wavelength and retention time at 260.0 nm over a 30min period. Figure 3 depicts the quantification levels of extracts of *Gypsophila* taxa.

The UPLC/UV method was used to concurrently quantify rosmarinic acid and rutin in the extracted samples of *Gypsophila* taxa. The results are presented in Table 5.

These results clearly demonstrate the UPLC/UV method’s effectiveness in accurately determining the rosmarinic acid and rutin levels in the *Gypsophila* extraction samples. Based on the rosmarinic acid quantification of the extracts, the *G. germanicopolitana* leaf extract had the highest quantity, followed by the *G. simonii* leaf extract and then the *G. germanicopolitana* stem extract. As for the rutin quantification of the extracts, the *G. simonii* leaf extract had the highest quantity, followed by the *G. germanicopolitana* leaf extract and then the *G. eriocalyx* leaf extract.

### 3.5. Antioxidant Capacity

The antioxidant capacity parameters (EC_50_ values) for extracts from *Gypsophila* taxa determined by the DPPH• and ABTS^+^•assaysare presented in Table 1. According to these results, the *G. eriocalyx* extract has the highest antioxidant capacity. The stem extract was revealed to have the highest capacity in the DPPH• assay (EC_50_ = 116.26 ± 0.07 µL extract), while the leaf extract showed the highest capacity in the ABTS^+^• study (EC_50_ = 11.85 ± 0.54). In all cases, the extracts obtained from the stem (S) showed a higher antioxidant capacity in the DPPH• assay, while extracts from the leaf (except for *G. germanicopolitana*) had a higher antioxidant capacity in the ABTS assay. Similar antioxidant activity is also evidenced by the FRAP assay, with the AAE value being highest in the case of the *G. eriocalyx* leaf extract (137.85 ± 0.34 µg AAE/mL) and lowest for the *G. simonii* stem extract (34.53 ± 0.25 µg AAE/mL).

The literature review on antioxidant activity revealed that different extracts of medicinal plants obtained by diverse methods and using various solvents were examined for their antioxidant capacity, which was expressed in different ways. However, comparing the values obtained for the tested species with the determined antioxidant capacities of nineteen medicinal plants [35], it is observed that endemic *Gypsophila* taxa have an average antioxidant capacity, thus demonstrating their potential for further investigation through invivo research in order to develop new pharmaceutical products.

### 3.6. In Vitro Evaluation of Carbonic Anhydrase Inhibition

In the body, enzymes act as important biocatalysts and can interact with all administered substances. Carbonic anhydrase (CA, *carbonate* dehydratases, E.C.4.2.1.1) is a drug-target enzyme involved in the regulation of pH and the transport of water, electrolytes, and ions. Also, CA catalyzes the hydration of CO_2_/dehydration of HCO_3_^−^ and the hydrolysis of many esters and the hydration of aldehydes, especially acetaldehyde. The inhibition or activation of CA is important in the treatment of many diseases such as diabetes, cancer, epilepsy, Alzheimer’s disease, and cardiovascular diseases. Therefore, determining the impact of plant extracts commonly used in alternative medicine on enzyme activity is essential.

All *Gypsophila* taxa extracts are assessed for their ability to inhibit the carbonic anhydrase enzyme in order to prove their usefulness as diuretic plants. Acetazolamide is a powerful inhibitor of CA and is used as a standard, displaying a well-defined linear calibration curve (Figure S2). The results for carbonic anhydrase inhibition and the equivalent acetazolamide concentration are presented in Table 1. The *G. germanicopolitana* leaf extract exhibits significant carbonic anhydrase inhibitory activity, with a value of 0.23 ± 0.01 mg/mL IC_50_ (Table 1) and 45.26 mg/mL equivalent to acetazolamide.

**Figure 4:**
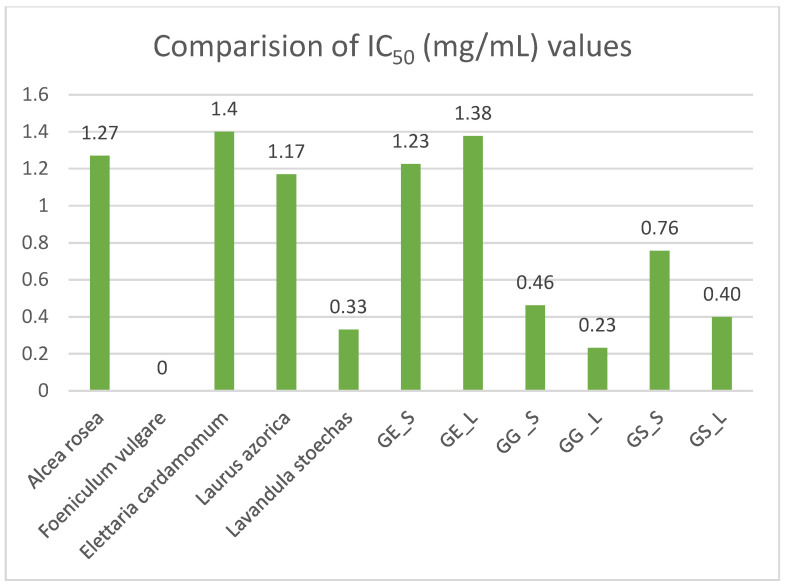
Comparison of in vitro diuretic activity of *Gypsophila* taxa and other diuretic plants [36] in terms of IC_50_ (mg/mL) values (GE_S: *G. eriocalyx* stem; GE_L: *G. eriocalyx* leaf; GG_S: *G. germanicopolitana* stem; GG_L: *G. germanicopolitana* leaf; GS_S: *G. simonii* stem; GS_L: *G. simonii* leaf).

The in vitro diuretic activity of the studied *Gypsophila* taxa was compared with data from the literature [34,36] for different diuretic plants, including *Alcea rosea*, *Foeniculum vulgare*, *Elettaria cardamomum*, *Laurusazorica*, and *Lavandula stoechas* (Figure 4). The comparison focused on their inhibition of the carbonic anhydrase enzyme, measured using IC_50_ values. Among the diuretic plant extracts, the leaf extract of *Gypsophila germanicopolitana* exhibited the highest carbonic anhydrase enzyme inhibition, with a value of 0.23 mg/mL.

### 3.7. Statistical Analysis of Results

**Figure 5:**
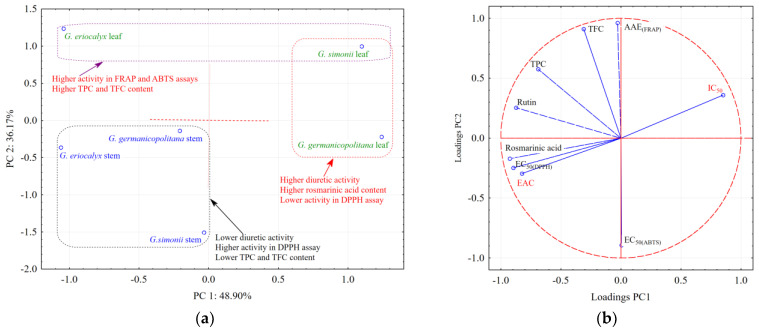
The PCA score plot of the experimental data illustrating the similarity/differentiation of the analyzed samples according to their determined parameters and biological activities (diuretic activity and antioxidant activity) (**a**) and the corresponding PCA loading plot of the first two PCs comparing the analyzed parameters (**b**).

Principal component analysis (PCA) was applied to show the samples’ classification and better reveal the parameters that are important in the diuretic activity of the analyzed *Gypsophila* taxa extracts. The score plot of the first two principal components (PC1 and PC2) (Figure 5a) revealed that the *G. germanopolitana* leaf and *G. simonii* leaf extracts are located together and associated with positive values for the PC1 scores and higher diuretic activity, higher rosmarinic acid and rutin content, and lower activity in the DPPH assay. On the other hand, the leaf extracts from *G. eriocalyx* and *G. simonii* are associated with positive values of PC2 scores and higher activity in the FRAP and ABTS assays and also a higher content of polyphenols and flavonoids (TPC and TFC). By this approach, the extracts from the stem, located together and associated with negative values of PC1 and PC2 scores, are characterized by lower diuretic activity, a lower content of polyphenols and flavonoids (TPC and TFC), and high activity in the DPPH assay.

Plot projection of the variables on the factor plane (PC1 × PC2) was used to find how the original variables and different biological activities (inhibition, diuretic, and antioxidant activity) are correlated which each other and how they are related to the principal components (PC1 and PC2). In this approach, the variables that are located together are positively correlated, while an angle of 180 degrees between variables means that they are inversely correlated. On the same principle, a small angle between variables indicates a positive correlation, while an angle of 90 degrees indicates no correlation.

According to the projection of the variables on the factor plane (Figure 5b), PC1 largely explains the variance related to diuretic and inhibition activities, while PC2 explains more of the variance associated with rutin, polyphenol, and flavonoid content. Rosmarinic acid is more correlated with the diuretic effect (direct correlation with EAC and inverse correlation with IC_50_ parameters) and antioxidant activity (EC_50(DPPH)_), since they are located together in the same direction (negatively along the PC1).

The significant correlations between the determined parameters are also supported by the statistical evaluation of the results outlined in Table 6.

Based on the obtained correlation, the in vitro diuretic activity is most affected by the presence of rosmarinic acid, followed by the quantity of rutin and the total polyphenol content. A significant correlation was observed between therosmarinic acid content and diuretic activity of the analyzed samples expressed both as the carbonic anhydrase inhibitory activity (IC_50_ values, correlation coefficient r = −0.95) and also as equivalent to acetazolamide (EAC values, correlation coefficient r = 0.93). According to the obtained statistically significant correlations, the rutin content is directly related to the antioxidant activity measured by the DPPH assay and to the polyphenol content (correlation coefficient r = 0.85 and r = 0.82),while the flavonoid content is directly related to the results from the FRAP assay (correlation coefficient r = 0.84). As mentioned by other studies [36], a significant correlation was revealed between the results from the FRAP and ABTS assays (r = −0.95). Moreover, this study revealed that the rutin, polyphenol, and flavonoid contents do not significantly contribute to the diuretic activity of the analyzed *Gypsophila* taxa extracts.

## 4. Conclusions

The present study reveals bio-metabolic profiles in the *Gypsophila* taxa, namely, *G. eriocalyx*, *G. germonicopolitana*, and *G. simonii*, finding for the first time the presence of rosmarinic acid and rutin in these plants. The in vitro diuretic activities of *Gypsophila* taxa are established for the first time. After the extraction process, the overall chemical composition in the ATR-FTIR spectra did not change greatly for both the stems and leaves. For this reason, the active compound types found in plants were not affected by the extraction process, and their structures were preserved. The change in band intensities in the spectra showed that the extraction process and leaf–stem parts contained different amounts of components.

The results showed that the stem extract of *G. eriocalyx* exhibited the highest antioxidant capacity determined by the DPPH method, while the leaf extract of *G. eriocalyx* showed the highest antioxidant capacity found by the ABTS assay. Additionally, the leaf extract of *G. eriocalyx* had the highest flavonoid content, and the leaf extract of *G. simonii* had the highest total polyphenolic content and the highest quantity of rutin. Furthermore, the leaf extract of *G. germanicopolitana* had the highest quantity of rosmarinic acid and the highest diuretic activity.

This study also revealed significant correlations between the rosmarinic acid content and diuretic activity and the DPPH• antioxidant capacity and rutin content, proving the fact that rosmarinic acid and rutin, even in low quantities, induce the diuretic activity and antioxidant capacity of the studied plants.

Likewise, it is recognized that the antioxidant capacity, phenolic compounds, and flavonoids have the capacity to augment the glomerular filtration rate through the amplification of renal blood flow, thereby promoting an escalation in urine production. Consequently, the pronounced diuretic effect of *Gypsophila* extracts may be attributed to the substantial flavonoid content and rosmarinic acid present in our extracts, especially in the case of the extract of *G. germanicopolitana* leaf. These findings will provide crucial insights into the potential therapeutic applications of *Gypsophila* taxa, particularly in treating diuretic-related ailments. Taking into account the promising results obtained, it is necessary to undertake further investigations to elucidate the underlying mechanism of action.

## Figures and Tables

**Table 1:** Carbonic anhydrase inhibition values expressed as IC_50_, equivalent acetazolamide concentration (EAC), total flavonoid content (TFC), total polyphenol content (TPC), and antioxidant capacity determined by DPPH, ABTS, and FRAP assays in extracts of *Gypsophila* taxa.

Species of *Gypsophila* Taxa	TFC (mg REs/mL ± SD)	TPC (mg GAEs/mL ± SD)	EC_50_ (µL Extract ± SD)		AAEs (µg/mL ± SD)	IC_50_ (mg/mL ± SD)	EAC (mg/mL ± SD)
			DPPH•Assay	ABTS^+^•Assay	FRAP Assay		
*G. eriocalyx* stem	0.204 ± 0.013	0.221 ± 0.0545	116.26 ± 0.07	13.67 ± 0.30	90.89 ± 0.38	1.23 ± 0.05	10.47 ± 0.21
*G. eriocalyx* leaf	0.642 ± 0.043	0.727 ± 0.027	125.91 ± 0.12	11.85 ± 0.54	137.85 ± 0.34	1.38 ± 0.87	6.21 ± 0.53
*G. germanicopolitana* stem	0.256 ± 0.033	0.451 ± 0.035	134.53 ± 0.02	14.31 ± 0.51	110.65 ± 0.04	0.46 ± 0.22	22.18 ± 0.32
*G. germanicopolitana* leaf	0.385 ± 0.021	0.613 ± 0.050	436.77 ± 0.27	16.50 ± 0.50	80.91 ± 0.54	0.23 ± 0.01	45.26 ± 0.82
*G. simonii* stem	0.072 ± 0.019	0.441 ± 0.042	341.03 ± 0.36	23.83 ± 1.88	34.53 ± 0.25	0.76 ± 0.05	15.09 ± 0.23
*G. simonii* leaf	0.524 ± 0.015	0.919 ± 0.039	476.20 ± 0.05	12.90 ± 0.55	119.06 ± 0.45	0.40 ± 0.09	24.37 ± 0.41

REs—rutin equivalents; GAEs—gallic acid equivalents; AAEs—ascorbic acid equivalents. SD—standard deviation calculated for results from three parallel samples.

**Table 2:** The analysis results, including the recovery values along with their corresponding mean, standard deviation (SD), and relative standard deviation (RSD) for rosmarinic acid, rutin, and quercetin.

Sample Number	Added (µg/mL)			Found (µg/mL)			Recovery (%)		
	Rutin	Rosmarinic Acid	Quercetin	Rutin	Rosmarinic Acid	Quercetin			
1	0.5	8.0	5.0	0.50	8.50	5.03	99.4	106.2	100.6
2	2.5	16	10	2.58	15.9	10.6	103.2	99.49	105.5
3	5.0	32	20	5.09	32.5	20.0	101.7	101.7	99.82
4	7.5	64	30	7.59	64.4	30.5	101.2	100.6	101.7
5	10	100	40	10.4	99.6	40.7	103.5	99.56	101.7
						Mean	101.5	101.9	101.9
						SD	1.65	2.19	2.19
						RSD	1.63	2.15	2.15

**Table 3:** The results for inter-day and intra-day analysis, including relative standard deviation (RSD) and relative standard error (RSE).

Experiment No	Added (µg/mL)			Found (µg/mL)			Recovery (%)		
	Rutin	Rosmarinic Acid	Quercetin	Rutin	Rosmarinic Acid	Quercetin	Rutin	Rosmarinic Acid	Quercetin
	Inter-day								
1	2.0	10	10	2.04	10.3	10.3	101.9	102.8	102.5
2	6.0	20	20	5.99	19.9	20.0	99.80	99.67	99.87
3	10	30	30	9.88	30.6	30.9	98.76	101.8	102.9
						**RSD (%)**	1.57	1.57	1.63
						**RSE (%)**	0.91	0.90	0.94
	**Intra-day**								
1	4.0	12	7.5	4.00	12.1	7.53	99.90	100.8	100.4
2	6.0	24	15	5.98	23.9	15.3	99.73	99.77	102.0
3	8.0	48	20	7.87	48.3	20.0	98.41	100.6	100.1
						**RSD (%)**	0.82	0.53	0.98
						**RSE (%)**	0.47	0.30	0.57

**Table 4:** The results of analysis for standard addition test samples.

Sample	Added (µg/mL)			Found (µg/mL)		
	Rutin	Rosmarinic Acid	Quercetin	Rutin	Rosmarinic Acid	Quercetin
0.1/10	4	20	12	4.07	19.95	12.49
0.1/10	8	40	18	8.08	40.75	18.05
0.1/10	10	80	24	10.04	80.05	24.38
				**Recovery (%)**		
				101.83	99.77	104.06
				100.94	101.89	100.3
				100.42	100.06	101.58
				**RSD (%)**		
				0.7	1.14	1.87

**Table 5:** The results of rutin and rosmarinic acid quantification in the extracts of *Gypsophila* taxa.

Species of *Gypsophila* Taxa	Concentration (µg/mL Extract)	
	Rutin	Rosmarinic Acid
*G. eriocalyx* stem	<LOD	2.40
*G. eriocalyx* leaf	0.01	1.36
*G. germanicopolitana* stem	<LOD	4.86
*G. germanicopolitana* leaf	0.03	7.38
*G.simonii* stem	0.01	3.04
*G. simonii* leaf	0.06	6.48

**Table 6:** Correlations of the determined parameters of the *Gypsophila* taxa extracts. Redbold-marked correlations are significant at *p* < 0.05 for confidence of 95%.

Determined Parameters	IC_50_	EAC	TFC	TPC	EC_50(DPPH)_	EC_50(ABTS)_	Rutin	Rosmarinic Acid
IC_50_	1.00	**−0.88**	0.12	−0.31	−0.73	−0.23	−0.54	**−0.95**
EAC		1.00	0.01	0.25	0.69	0.13	0.48	**0.93**
TFC			1.00	0.79	0.09	−0.74	0.49	0.08
TPC				1.00	0.58	−0.32	**0.82**	0.41
EC_50(DPPH)_					1.00	0.33	**0.85**	0.76
EC_50(ABTS)_						1.00	−0.16	0.01
Rutin							1.00	0.68
Rosmarinic acid								1.00

## Data Availability

Data is contained within the article and Supplementary Material.

## Supplementary Materials

The following supporting information can be downloaded at https://www.mdpi.com/article/10.3390/antiox14020219/s1: Figure S1: Chromatogram of standard compounds (rosmarinic acid, rutin, coumaric acid, quercetin, apigenin, and kaempferol)obtained using optimized method for the mixture solutions (each compound was 40 ppm). Figure S2: Linear calibration curve of Acetazolamide. Figure S3: Rutin calibration curve. Figure S4: Gallic acid calibration curve.

## Abbreviations

The following abbreviations are used in this manuscript:

AAEs	Ascorbic acid equivalents
ABTS•+	2,2′-azinobis-(3-ethylbenzothiazoline-6-sulfonic acid)
DPPH•	2,2-diphenyl-1-picrylhydrazyl
CA	Carbonic anhydrase (*carbonate* dehydratases, E.C.4.2.1.1)
EC_50_	Efficient concentration
EAC	Equivalent to acetazolamide
PC1 vs. PC2	First two principal components
FRAP	Ferric reducing antioxidant power
FT-IR	Fourier-transform infrared spectroscopy
GAEs	Gallic acid equivalents
HPLC	High-pressure liquid chromatography
IUCN	International union for conservation of nature
IC_50_	Inhibition concentration
PCA	Principal component analysis
TFC	Total flavonoid content
TPC	Total polyphenol content
TPTZ	2,4,6-tripyridyl-s-triazine
UPLC	Ultra-pressure liquid chromatography
UV	Ultraviolet
