# Supplementary material for: Unveiling the Potential of Three Endemic Gypsophila L. (Caryophyllaceae) Taxa as Promising Carbonic Anhydrase Inhibitors—Bio-Metabolic Profiles and In Vitro Evaluation of Enzyme Inhibition and Antioxidant Capacity
Source: Antioxidants (Basel). 2025 Feb 14;14(2):219. doi: 10.3390/antiox14020219 (PMC11852014; doi:10.3390/antiox14020219)
Supplement: Supplementary file 1 [file antioxidants-14-00219-s001.zip › antioxidants-3462495-supplementary.pdf]

Supplementary material

Physiology and Molecular Biology of Plants

Unveiling the potential of three endemic Gypsophila L. (Caryophyllaceae) as promising carbonic anhydrase inhibitors - bio-metabolic profiles and *in vitro* evaluation of enzyme inhibition and antioxidant capacity

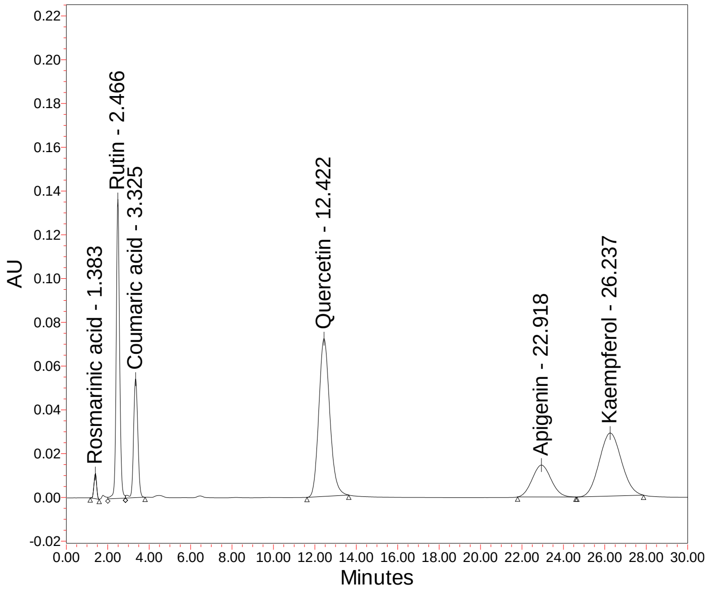

Figure S1 Chromatogram of standard compounds (rosmarinic acid, rutin, coumaric acid, quercetin, apigenin, and kaempferol, respectively)obtained using optimized method in the mixture solutions (each compound was 40 ppm)

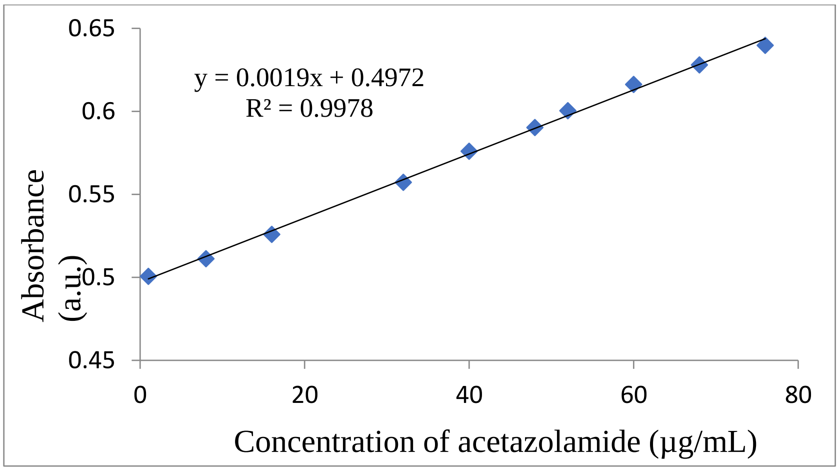

Figure S2 Linear calibration curve of Acetazolamide

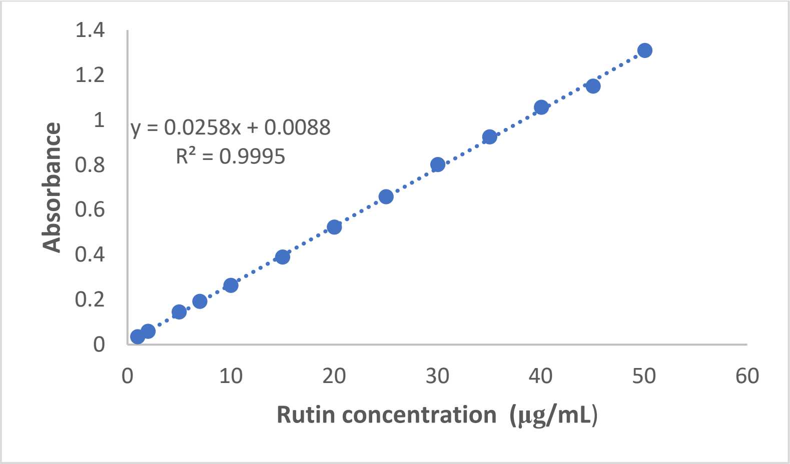

Figure S3 Rutin calibration curve

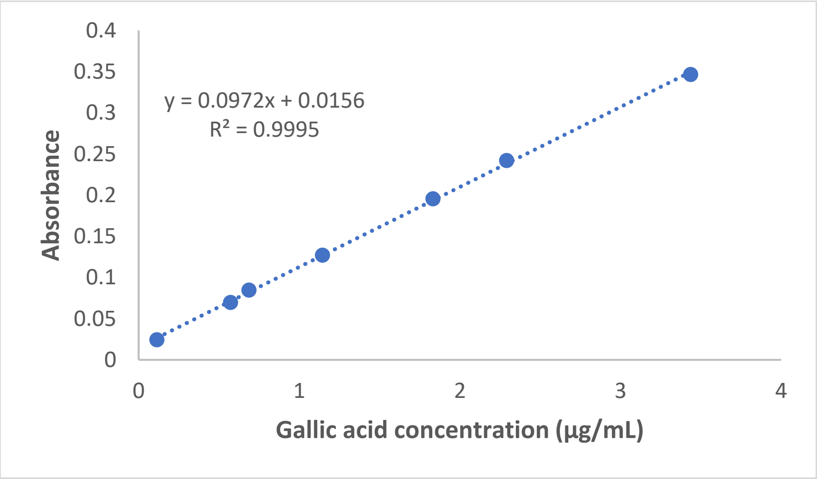

Figure S4 Gallic acid calibration curve
